# The role of pericyte in ocular vascular diseases

**DOI:** 10.7555/JBR.37.20230314

**Published:** 2024-05-29

**Authors:** Lianjun Shi, Huimin Ge, Fan Ye, Xiumiao Li, Qin Jiang

**Affiliations:** 1 The Affiliated Eye Hospital of Nanjing Medical University, Nanjing, Jiangsu 210029, China; 2 The Fourth School of Clinical Medicine, Nanjing Medical University, Nanjing, Jiangsu 210029, China

**Keywords:** pericyte, vascular stability, vascular diseases, diabetic retinopathy, choroidal neovascularization

## Abstract

Pericytes are located in the stromal membrane of the capillary outer wall and contain endothelial cells. They are pivotal in regulating blood flow, enhancing vascular stability, and maintaining the integrity of the blood-retina barrier/blood-brain barrier. The pluripotency of pericytes allows them to differentiate into various cell types, highlighting their significance in vascular disease pathogenesis, as demonstrated by previous studies. This capability enables pericytes to be a potential biomarker for the diagnosis and a target for the treatment of vascular disorders. The retina, an essential part of the eyeball, is an extension of cerebral tissue with a transparent refractive medium. It offers a unique window for assessing systemic microvascular lesions. Routine fundus examination is necessary for patients with diabetes and hypertension. Manifestations, such as retinal artery tortuosity, dilation, stenosis, and abnormal arteriovenous anastomosis, serve as typical hallmarks of retinal vasculopathy. Therefore, studies of ocular vascular diseases significantly facilitate the exploration of systemic vascular diseases.

## Introduction

Pericytes are one of the mural cells of microvessels, located within the capillary basement membrane, wrapping around endothelial cells (ECs). Pericytes are thought to be tightly associated with ECs and contribute to the proliferation and differentiation of ECs ^[ 1] ^. Thus, pericytes play a critical role in vascular morphogenesis, maturation, and stability ^[ 2] ^. Moreover, the contractile characteristic of pericytes enables them to regulate vascular diameter and blood flow ^[ 3] ^. On the other hand, without pericyte recruitment, ECs fail to deposit into stable basement membranes, and neovascular vessels appear to be wide and short over time; at the same time, pericyte recruitment and proliferation in angiogenesis require the participation of ECs ^[ 4] ^. The loss or dysfunction of pericytes may result in the onset and progression of various diseases, such as cancer ^[ 5– 6] ^, neurodegenerative diseases ^[ 7] ^, and fibrotic diseases ^[ 8] ^.

Pericyte density is higher in the retina than in other tissues, involved in more active pericyte-EC interactions and more complex paracrine activity. Pericyte-endothelial interactions are essential for the integrity and functionality of the retinal neurovascular unit. However, oxidative stress and other pathological conditions may disrupt the pericyte-EC interactions, leading to abnormal retinal microvasculature ^[ 9] ^. Recent evidence has indicated that pericytes play a role in several ocular diseases, such as diabetic retinopathy (DR) ^[ 10] ^, choroidal neovascularization ^[ 11] ^, corneal neovascularization ^[ 12] ^, retinopathy of prematurity ^[ 13] ^, and glaucoma ^[ 14] ^.

Although it is generally acknowledged that pericytes are a sublineage of vascular smooth muscle cells, a specific and consistent molecular marker of pericytes is still missing ^[ 15] ^. Pericytes distributed in different organs may differ in morphology, molecular markers, and biological functions, but there is no consensus on clear criteria for pericyte differentiation, leading to the identification of pericytes remaining a puzzle. Therefore, in the current review, we elaborate on biological functions of the pericytes and their roles in ocular vascular diseases, aiming to identify potential therapeutic targets for the treatment of ocular vascular disease.

## Pericytes in microvasculature

**Figure 1:**
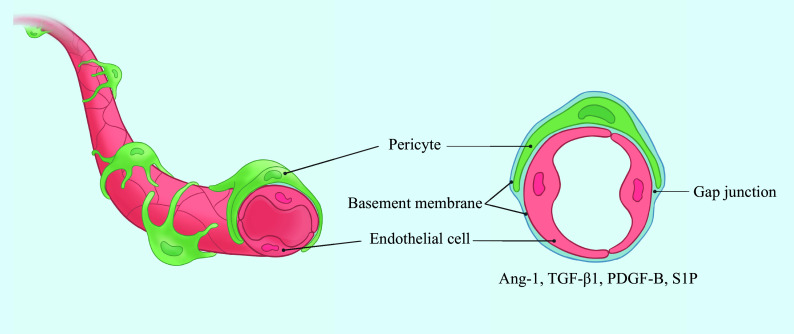
Position relationship between pericytes and ECs. Pericytes are embedded in the basal membrane of capillary endothelial cells and communicate with endothelial cells *via* gap junctions at peg-sockets and other paracrine signaling factors. Abbreviations: Ang-1, angiopoietin-1; TGF-β1, transforming growth factor-β1; PDGF-B, platelet-derived growth factor B; S1P, sphingosine-1-phosphate.

Pericytes are adjacent to ECs within the microvasculature ( ***Fig. 1***), and are found in about 80% of structures in the systemic microcirculation, such as arterioles, capillaries, and venules ^[ 16] ^. The contractility of pericytes is similar to that of smooth muscle cells in arterioles and great vessels. Pericytes, which originate from diverse origins spanning from mesoderm to neuroectoderm, differentiate into various mesenchymal cell types, including adipocytes, chondrocytes, osteoblasts, fibroblasts, and vascular smooth muscle cells. Interacting with ECs through gap junctions and a shared basement membrane ^[ 17] ^, pericytes assist in maintaining vascular permeability, regulating blood vessel diameter, and preventing intercellular edema. These effects are mediated by their interaction with angiotensin, transforming growth factor-β1 (TGF-β1), platelet-derived growth factor B (PDGF-B), and sphingosine-1-phosphate (S1P). Notably, the retinal vascular network contains an abundant quantity of pericytes and exhibits a more active EC-pericyte interaction, compared with cerebral tissues ^[ 18– 20] ^. Additionally, the retinal vascular network shows a superior efficiency in transporting small molecules, such as glucose ^[ 21] ^.

Pericytes express diverse markers, including intracellular contractile and cytoskeletal proteins (such as α-smooth muscle actin [α-SMA], vimentin, desmin, myosin, and nestin), membrane proteins (such as neural/glial antigen 2 [NG2], platelet-derived growth factor receptor β [PDGFRβ], cluster of differentiation 31 [CD31], regulators of G protein signaling 5, and cluster of differentiation 146 [CD146]) ^[ 6, 22] ^, as well as surface markers with properties of mesenchymal stem cells, which facilitate their differentiation into adipocytes, osteoblasts, and chondrocytes ^[ 23] ^. However, pericytes lack markers of vascular ECs (such as CD31 and von Willebrand factor [VWF]), glial cells (such as glial fibrillary acidic protein [GFAP] and oligodendrocyte lineage transcription factor 2 [OLIG2]), microglial cells (ionized calcium-binding adapter molecule 1), neurons, and paravascular cells. Although no specific markers for pericytes exist, the combination of NG2 and PDGFRβ is commonly used to identify specific subgroups of pericytes ^[ 24– 25] ^, particularly within capillaries, postcapillary venules, and terminal arterioles ^[ 26] ^.

## Biological functions of pericytes

Pericytes exhibit varying genetic backgrounds and functions, because of their different origins. For instance, pericytes in the brain are crucial in maintaining the permeability and integrity of the blood-brain barrier (BBB), regulating the entry of small molecules into cerebral tissues ^[ 27] ^. Liver-specific pericytes bear functions such as vitamin A storage, recruitment of inflammatory cells for tissue repair, and extracellular matrix remodeling ^[ 28] ^. CD146 ^+^ pericytes derived from the skeletal muscles, on the other hand, exhibit significant myogenic activity and regeneration potential, similar to muscle cells ^[ 29] ^.

The multifunctional nature of pericytes is detailed as follows: Ⅰ. Angiogenesis promotion and vascular stability maintenance. Pericytes are crucial regulators for revascularization, microvascular stability, central nervous system development, and vascular remodeling ^[ 24] ^. Ⅱ. Participating in blood flow regulation and coupling of neurovascular functional units. In the brain and retinal tissues, pericytes contract or relax under the action of vasoconstrictor factors (adrenergic agonists, histamines, serotonin, angiotensin Ⅱ, and endothelin-1) and vasodilator factors (NO and cholinergic agonists), regulating vascular tone and lumen diameter through the arachidonic acid metabolic pathway, modulating microcirculation blood flow ^[ 3, 30] ^. The role of pericytes in blood flow regulation, particularly in medium blood vessels, remains debated, with some investigators suggesting a less defined role ^[ 31– 32] ^. Ⅲ. Key components of BBB/blood-retinal barrier (BRB). Under physiological conditions, intercellular connexins, which are highly expressed, foster meaningful interactions between ECs and pericytes to maintain barrier integrity and stability ^[ 17] ^. In contrast, the loss of pericytes destroys BBB and BRB structures, marked by a reduced expression of intercellular tight junction proteins and an increased vascular leakage. These findings highlight the critical role of pericytes in the transport system of BBB and BRB ^[ 9, 33] ^. Ⅳ. Neuroinflammatory regulation. Pericytes function as perivascular macrophages to clear tissue debris and foreign antigens. They secrete intercellular adhesion molecule-1 (ICAM-1) to facilitate leukocyte adhesion and migration *via* binding to integrin ligands on leukocytes ^[ 34] ^. In the Alzheimer's model, pericytes participate in amyloid β-protein clearance in the retina and central nervous system ^[ 35– 36] ^. Notably, in pericyte-deficient mice, leukocyte adhesion and migration mediated by ECs were found to be defective, indicating their role in inflammatory responses ^[ 37] ^. Ⅴ. Stem cell-like property. Pericytes exhibit a significant differentiation potential *in vitro*. Following cerebral ischemia induction in adult mice, pericytes sourced from the brain tissues demonstrated the ability to differentiate into neural and vascular cell lines ^[ 23] ^. Coronary microvascular pericytes may differentiate into smooth muscle cells *via* activating the Notch signaling pathway ^[ 38] ^. Additionally, pericytes may also differentiate into non-mesenchymal cells, such as neurons. Notably, pericytes derived from skeletal muscles have been reported to differentiate into the high-class Ⅲ beta-tubulin-expressing neurons ^[ 39] ^.

## Pericytes and vascular dysfunction

**Figure 2:**
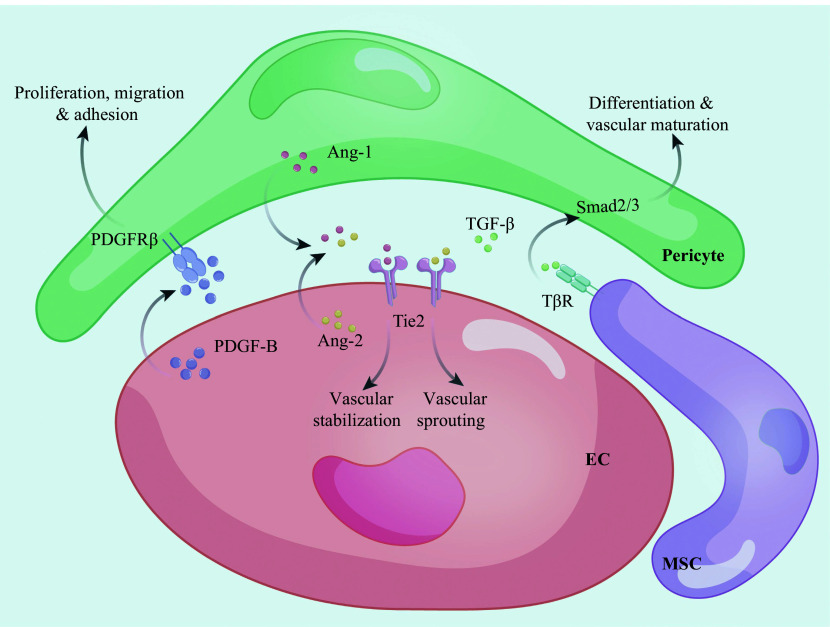
Three main signaling pathways of the pericyte-EC regulation. The PDGF-B/PDGFRβ axis promotes the proliferation, migration, and adhesion of pericytes. The Ang/Tie2 axis regulates vascular sprouting and vascular stabilization. The TGF-β/TβR axis regulates pericyte differentiation and vascular maturation through Smad2/3 and downstream target genes. Abbreviations: EC, endothelial cell; MSC, mesenchymal stem cell; PDGF-B, platelet-derived growth factor B; PDGFRβ, platelet-derived growth factor receptor β; Ang-1, angiopoietin-1; Ang-2, angiopoietin-2; TGF-β, transforming growth factor-β; TβR, TGF-β receptor.

Pericytes participate in vascular budding by interacting with vascular ECs ^[ 2, 40] ^, as depicted in ***Fig. 2***. Through intercellular connections, pericytes regulate the migration, proliferation, permeability, and contractility of vascular ECs *via* the paracrine signaling.

ECs recruit pericytes and adhere to the vascular wall by regulating the PDGF signaling pathway. The PDGF-B/PDGFRβ signal transduction pathway plays a pivotal role in pericyte proliferation, migration, apoptosis, and adhesion. PDGF-B, secreted from vascular ECs, interacts with PDGFRβ on pericytes, prompting their chemotaxis and adhesion to the outer wall of neovessels. Disruption of the PDGF-B/PDGFRβ signaling pathway impairs pericyte functions mentioned above, reduces their coverage around ECs, and induces aberrant EC proliferation, abnormal vascular morphology, and microhemangioma ^[ 41– 42] ^. Moreover, blocking the PDGF signaling pathway induces pericyte death, which hampers the recruitment of pericytes towards vascular ECs, weakens inter-endothelial barriers, and increases macromolecule leakage into the extracellular matrix. Consequently, disruption of the PDGF signaling pathway triggers inflammation and pathological remodeling of systemic tissues and organs. The PDGF-B knockout in vascular ECs leads to irregular distribution or loss of pericytes, BRB disruption and retinal vascular anomalies ^[ 43] ^. However, PDGF-B overexpression in photoreceptor cells induces proliferation of pericytes, astrocytes, and ECs, and then forms irregular cell sheaths and clumps. These structures subsequently migrate to the inner retina, leading to the retinal traction and ultimately causing the retinal detachment. Crucially, PDGF-B overexpression inhibits the development of deep retinal vessels in mice, highlighting its pivotal role in pericyte density regulation in retinal tissues ^[ 44] ^. Importantly, normal secretion of PDGF-B by ECs is essential for retinal vascular development and homeostasis. The angiopoietin-1 (Ang-1)/Tie2 signaling pathway is also essential for vascular maturation and stability. Disruption of this pathway, either through *Ang1* knockout or Tie2 antagonism, significantly increases pericyte loss; however, *Tie2* knockout results in aberrant angiogenesis and diverse vascular lumen sizes during embryonic development, ultimately causing embryonic death ^[ 45] ^. Additionally, TGF-β interaction with transforming growth factor-β type 1 receptor (TGFβR1) on mesenchymal stem cells triggers Smad transcription factors 2 and 3 activation, promoting the differentiation of mesenchymal stem cells into pericytes and vascular maturation ^[ 46] ^.

These three signal transduction pathways synergistically modulate pericyte functions, thus modulating the processes in vascular diseases. In addition, ECs recruit pericytes through various ligand-receptor interactions, such as stromal cell-derived factor 1/C-X-C chemokine receptor 4, heparin-binding epidermal growth factor/ErbB, and Sonic hedgehog protein (Shh)/Ptc ^[ 47– 48] ^. Moreover, non-coding RNAs are also involved in the regulation of pericyte functions, adding complexity to the molecular interplay in vascular diseases. A study by Demolli *et al* ^[ 48]^ highlights the role of miR-27 in enhancing EC-pericyte interaction *via* semaphorin 6A and 6D upregulation. The increased pericyte proliferation in damaged and fibrotic tissues has been shown to benefit tissue repair and organ remodeling ^[ 49] ^, while long non-coding RNA *TYKRIL* promotes pericellular proliferation *via* PDGFRβ, and its inhibition has been shown to reverse pulmonary hypertension ^[ 50] ^, underscoring the significance of pericytes in vascular disease pathogenesis.

## The role of pericytes in ocular vascular diseases

Pericytes are increasingly recognized for their critical roles in the pathogenesis of various ocular vascular diseases, including DR, choroidal neovascularization, corneal neovascularization, and retinopathy of prematurity.

### DR

DR initially manifests as hyperglycemia-induced pericyte apoptosis and loss, leaving only vacuole-like structures on the vascular wall. The resultant pericyte apoptosis disrupts EC proliferation, exacerbates vascular leakage, and triggers inflammatory responses, thereby destroying the BRB integrity. This disruption precipitates pathological vascular proliferation and retinal degeneration ^[ 51] ^. The retina at the injured site shows punctate hemorrhage and yellowish-white deposition ( *i.e.*, hard exudates). As DR progresses, the complete destruction of terminal microvascular structures causes the formation of acellular capillaries, precipitating local retinal tissue ischemia and hypoxia. In response, compensatory capillary dilation at the peripheral lesions occurs, manifesting clinically as microhemangiomas that represent the earliest detectable pathological change in DR.

**Figure 3:**
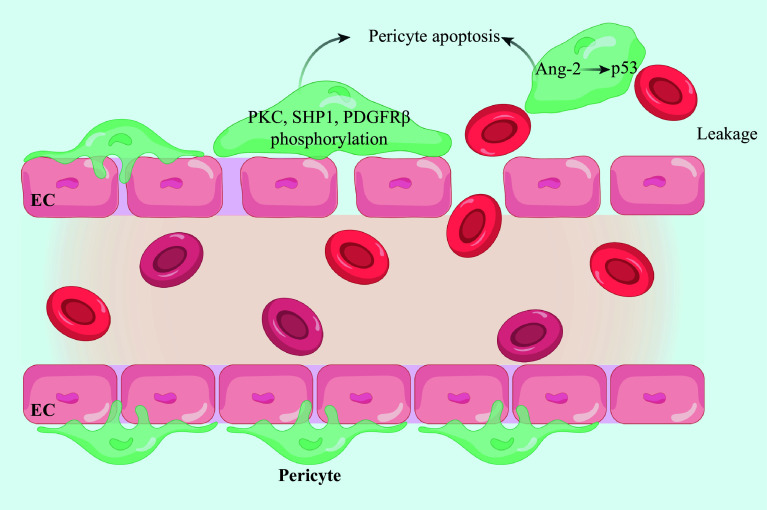
High glucose leads to the loss of pericytes and vascular leakage. Hyperglycemia promotes the phosphorylation of intracellular tyrosine proteins, such as PKC, SHP1, and PDGFRβ, in pericytes, thus promoting pericyte apoptosis. Ang-2 in pericytes activates apoptosis through the p53 signal transduction pathway. Abbreviations: EC, endothelial cell; Ang-2, angiopoietin-2; PKC, protein kinase C; SHP1, Scr homology-2 domain-containing phosphatase 1; PDGFRβ, platelet-derived growth factor receptor β.

Prolonged ischemia and hypoxia in retinal tissues trigger pathological neovascularization, which is a hallmark of proliferative diabetic retinopathy (PDR), highlighting the transition into the proliferative stage of the disease. PDR is characterized by fragile and incomplete vascular structures that predispose the patient to hemorrhage. Bleeding within the vitreous cavity will induce fibrosis, and subsequent contraction of the vascular membranes leads to retinal traction, potentially causing retinal tear or even detachment ^[ 52] ^. These pathological changes, if untreated, may result in irreversible blindness, underscoring the imperative for designing timely intervention and robust management strategies targeting PDR. Hyperglycemia, advanced glycation end-products (AGEs), stromal membrane thickening, and hypertension are essential in the pathogenesis of pericyte apoptosis in DR. In the progression of DR, pericyte apoptosis is considered the initial trigger to set off serial pathological processes, which is evoked by hyperglycemia-induced phosphorylation of intracellular tyrosine proteins, such as protein kinase C, small heterodimer partner 1, and PDGFRβ, leading to the subsequent dephosphorylation and inactivation of anti-apoptotic intracellular signaling molecules ^[ 53– 54] ^, as illustrated in ***Fig. 3***. It has been reported that angiopoietin-2 (Ang-2) within pericytes triggers apoptosis *via* the p53 signal transduction pathway in the retina of diabetic mice, and the intravitreal injection of Ang-2 in normal mice similarly induces pericyte apoptosis, whereas *Ang2* knockout significantly inhibits the apoptotic outcome ^[ 55] ^. Another study has reported a significant reduction in the pericyte/EC ratio within the DR vascular tissues ^[ 17] ^, and our previous study also revealed that pericyte depletion impaired biological functions of ECs *via* exosomes carrying circRNA-PWWP2A in DR ^[ 56] ^.

In the advanced stages of PDR, the exacerbation of ischemia and hypoxia leads to further pericyte loss, which induces the destabilization of pathological neovascularization, thus increasing susceptibility to vascular leakage and hemorrhage ^[ 57] ^. Consequently, protecting pericytes and their functional stability is crucial in DR treatment. The transplantation of vascular smooth muscle cells, capable of differentiating into pericytes, significantly repairs retinal vascular damage and lessens vascular leakage in DR model rats ^[ 58] ^. Complementing these findings, our previous study demonstrated that the upregulation of circRNA-ZNF532 or the inhibition of miR-29a-3p effectively ameliorated the retinal pericyte degeneration and vascular dysfunction in the vitreous body induced by diabetes ^[ 18] ^, in which we found that pericytes sourced from normal mice exhibited a significantly superior vascular repair capacity, compared with those from their diabetic counterparts, suggesting that enhancing the function and quantity of autogenous pericytes is essential in treating retinal vascular diseases. These findings collectively reveal the pivotal role of pericytes in DR progression and suggest that therapies targeting pericytes may offer a promising choice for managing diabetic microvascular complications.

### Choroidal neovascularization (CNV)

Age-related macular degeneration (AMD), a leading cause of blindness among the elderly, is usually divided into dry AMD and wet AMD. Wet AMD primarily manifested the damage of retinal pigmentation epithelium cells and CNV ^[ 59] ^, and CNV stands as the hallmark pathological feature of wet AMD. Pathological evaluations of ocular CNV lesions in AMD patients have revealed alterations in the quantity and morphology of pericytes. A comparative analysis of CNV lesions across varying AMD stages demonstrates a greater loss of pericytes in the advanced AMD cases, compared with those in early stages. Subretinal fibrosis, a critical AMD indicator, is linked to pericyte-to-myofibroblast transformation ^[ 60] ^. In a rodent model of laser-induced CNV, it was shown that the exogenous introduction of hematopoietic stem cells differentiated into pericytes in the injured regions, correlating positively with vascular maturation ^[ 61] ^. In alignment with these findings, treatment with axitinib significantly reduced pericyte migration towards CNV lesions, resulting in pathological neovascularization leakage and inducing degeneration *in vivo* ^[ 62] ^. Additionally, EYE-101, a novel retinoid-based drug, showed the efficacy of reducing choroidal sprouting and suppressing laser-induced CNV formation by decreasing pericyte coverage on ocular blood vessels ^[ 63] ^, highlighting that the quantity and morphology of pericytes exert significant effects on the progression of CNV diseases.

### Corneal neovascularization

The cornea, the transparent avascular tissues under physiological conditions, plays a crucial role in the ocular refractive system. However, exposure to pathological stimuli may trigger neovascularization within the corneal tissues, aiding in regressing the inflammatory response, repairing damaged tissues, and inhibiting the lysis of corneal tissues ^[ 64] ^. Destruction in the inherent structural stability and transparency of the corneal tissues by neovascularization is a leading cause of corneal blindness ^[ 65] ^. Cursiefen *et al* ^[ 66]^ reported that, within two weeks of human corneal neovascularization, 80% of the vascular tissues were strewn with pericytes, suggesting their role in the pathological process of corneal neovascularization. Additionally, pericytes in corneal neovascular tissues originated not only from the differentiation of bone marrow hematopoietic stem cells but also from limbal stem cells ^[ 67] ^. Notably, bone marrow-derived pericytes contribute to corneal neovascularization and lymphangiogenesis. Another study by Xu *et al* ^[ 60]^ has revealed the participation of pericytes in corneal neovascularization, particularly noting their relationship with vascular leakage in the corneal tissues of the corneal alkali burn mouse model. Inhibition of the PDGF signaling pathway leads to pericyte loss and vascular density reduction within the neovascularized cornea ^[ 68] ^. Additionally, NG2 antagonists may significantly reduce the onset and progression of pathological neovascularization in the cornea ^[ 69] ^. Therefore, pericyte dysfunction may exacerbate corneal pathological neovascularization, and targeting pericytes may provide a new direction for the future treatment of corneal neovascularization-related diseases.

### Retinopathy of prematurity (ROP)

Premature infants are particularly vulnerable to severe complications, such as retinal neovascularization and retinal detachment, which often develop in pathological conditions, such as oxygen poisoning and secondary hypoxia ^[ 70] ^. ROP, a primary cause of blindness in children, is characterized by retinal neovascularization induced by fluctuating levels of oxygen concentration. Oxygen-induced retinopathy has often been used in animal models for ROP research, in which the *Pdgfb* knockout significantly increased pericyte loss in retinal tissue, with a double incidence of neovascularization, compared with the control mice ^[ 71] ^. One study showed that PDGFRβ inhibitors elicited pericyte apoptosis, upregulated vascular endothelial growth factor (VEGF)/VEGF receptor expression, and consequently accelerated neovascularization ^[ 72] ^. Another study demonstrated that intravitreal injection of VEGF in lactating mice aged 4–6 days reduced apoptosis of vascular ECs and pericytes under hypoxic conditions, concurrently decreasing the extent of vascular occlusion ^[ 73] ^. These findings suggest that the diminished pericyte coverage, under activation of the VEGF signaling transduction pathway, may promote the budding of new retinal vessels in hypoxic environments. Additionally, Fukushi *et al* ^[ 72]^ demonstrated that soluble NG2 molecules enhanced the motility of vascular ECs and facilitated vascular cord formation *in vitro*, highlighting the dual role of pericytes in neovasculature by promoting vascular stability and neovascular proliferation. Another study showed that silencing *Col1a1*, a marker gene of pericyte subpopulation 2, not only reduced the neovascular and avascular regions in the retinas of oxygen-induced retinopathy but also suppressed pericyte-myofibroblast transition, underlining the significance of pericyte heterogeneity in pathological angiogenesis at the single-cell level ^[ 74] ^. Collectively, these studies emphasize the significant functions of pericytes in modulating hypoxic-ischemic retinal vascular diseases.

### Glaucoma

Glaucoma ranks as a leading cause of irreversible blindness worldwide. It remains incurable, with current treatments largely focusing on managing intraocular pressure ^[ 75] ^. The pathophysiology of glaucoma is primarily characterized by the gradual loss of retinal ganglion cells (RGCs). RGCs are the only output neurons transmitting visual signals from the retina to the brain. RGCs require a rich blood supply of oxygen and nutrients because of their high metabolic activity ^[ 76] ^. However, in glaucoma patients, vascular abnormalities are common and result in a decreased blood flow and an impaired neurovascular coupling ^[ 77] ^. Retinal pericytes, equipped with contractile proteins, play a key role in microcirculatory flow regulation *via* adjusting capillary diameters ^[ 78] ^. A previous study discovered that two types of bona fide pericytes, from distinct capillary systems, connected together to form a functional network termed interpericyte tunneling nanotubes (IP-TNTs) ^[ 79] ^. These structures linked two bona fide pericytes *via* CX43 gap junctions, allowing the transfer of ions and small molecules, while keeping their cytoplasm separated ^[ 79] ^; however, IP-TNTs were susceptible to damage from ischemic and glaucomatous conditions, through mechanisms involving excessive calcium influx into pericytes. This is because pericyte-specific inhibition of excessive Ca ^2+^ influx protects IP-TNTs and neurovascular coupling, ultimately preserving retinal neuronal function in glaucoma ^[ 75] ^. Debate persists regarding the neurovascular interactions in glaucoma. A prevailing view suggests that damage to RGCs leads to imbalanced delivery of neuronal-derived products upon pericyte receptors, potentially resulting in vascular dysfunction and impaired blood delivery, which exacerbate injury ^[ 14] ^. However, recent studies have demonstrated that signals from pericytes/capillaries to RGCs also contribute to the pathology of neurovascular coupling, suggesting that pericyte/capillary dysfunction directly may impair neuronal activity and potentially compromise RGC viability in glaucoma ^[ 75] ^. In summary, the mechanisms underlying these observations warrant future investigation.

## Conclusions and perspectives

Pericytes, as critical components of the vascular wall, are pivotal in the pathogenesis of vascular diseases. An increasing number of studies have indicated that pericyte loss reduces vascular structural stability and heightens susceptibility to vascular regulatory factors in the microenvironment in ocular vascular diseases. Furthermore, pericyte loss disrupts vascular EC function and alters vascular morphology. Therefore, future studies should focus on exploring the mechanisms of pericyte-mediated vascular homeostasis, regulation of EC function, and cell apoptosis under pathological conditions. Future pericyte-targeting therapies hold a significant promise. Strategies aimed at enhancing pericyte activity, reducing pathological loss, and providing effective exogenous supplements are expected to revolutionize clinical management of vascular diseases.
